# Polyamidoamine (PAMAM) Dendrimers Modified with Cathepsin-B Cleavable Oligopeptides for Enhanced Gene Delivery

**DOI:** 10.3390/polym9060224

**Published:** 2017-06-14

**Authors:** Seulgi Lee, Sang Jae Son, Su Jeong Song, Tai Hwan Ha, Joon Sig Choi

**Affiliations:** 1 Department of Biochemistry, Chungnam National University, Daejeon 305-764, Korea; smileleesg@gmail.com (S.L.); song.sj87@gmail.com (S.J.S.); 2 Hazards Monitoring BNT Research Center, Korea Research Institute of Bioscience and Biotechnology (KRIBB), 125 Gwahak-ro, Yuseong-gu, Daejeon 34141, Korea; 3 Graduate School of Analytical Science and Technology, Chungnam National University, Daejeon 34134, Korea; sson7911@naver.com; 4 Department of Nanobiotechnology, KRIBB School of Biotechnology, Korea University of Science and Technology (UST), 217 Gajeong-ro, Yuseong-gu, Daejeon 34113, Korea

**Keywords:** gene delivery, polyplex, poly(amidoamine) dendrimer, GFLG peptide, cathepsin B, histidine, arginine

## Abstract

Because of the complex mechanisms mediating cancer onset, prognosis, and metastatic behavior, different therapeutic approaches targeting these mechanisms have been investigated. Recent advancements in nanocarrier-based drug and gene delivery methods have encouraged scientific groups to investigate various novel therapeutic techniques. In this study, a poly(amidoamine) (PAMAM) polymer-based gene carrier containing the cathepsin B-enzyme sensitive sequence (glycine-phenylalanine-leucine-glycine, GFLG) was evaluated to determine transfection efficiency. Following the GFLG sequence, the surface of PAMAM generation 4 (G4) was conjugated with histidine (H) and arginine (R) for improved endosomal escape and cellular uptake, respectively. The successful synthesis of G4-GLFG-H-R was confirmed by ^1^H-nuclear magnetic resonance spectroscopy. The polyplex composed of G4-GLFG-H-R and pDNA was simulated by the enzyme cathepsin B and induced endosomal escape of pDNA, which was confirmed by gel electrophoresis. Compared with the G4 control, enzyme-sensitive G4-GLFG-H-R showed higher transfection efficiency and lower cytotoxicity in HeLa cells. These results demonstrated that G4-GLFG-H-R may be a highly potent and efficient carrier for gene therapy applications.

## 1. Introduction

Efficient cancer therapy is urgently needed because of the disease’s complicated onset and growth mechanisms and the increasing incidence rates of various types of cancer. Recent cancer statistics have revealed that many types of cancers, including breast, melanoma, prostate, and colon cancer, have unknown mechanisms of onset and resistance to conventional therapies [1]. However, the number of cancer survivors has increased because of early detection, leading to improved treatment and prevention. Promising treatments such as immunotherapy, nanoparticle-based targeted therapy, and combination therapies are currently undergoing clinical trials [2,3].

Gene delivery is a type of cancer therapy that uses nucleic acids to replace defective or missing genes, or to restore or turn off a specific gene function [4]. For effective gene delivery, viral and non-viral vectors have been extensively evaluated over the past few decades [5]. However, while viral vectors have high transfection efficiency, their immunogenicity is a significant obstacle. In contrast, non-viral vectors offer various advantages over viral vectors, including low cytotoxicity, tunable size and surface properties, and the ability to condense larger nucleic acids for intracellular delivery [6,7,8]. Therefore, despite their low transfection efficiency, novel strategies are still being investigated to maximize efficacy.

To overcome the shortcomings of non-viral vectors, various chemical conjugation or physical transformations have been attempted. To date, liposomes, polymer-based carriers such as cationic polymers and self-assembled micelles have been proven to be the most efficient and to have the prerequisite characteristics for gene therapy [9,10].

The poly(amidoamine) (PAMAM) dendrimer is an ideal cationic polymer with an ethylene diamine (EDA) core, abundant primary and tertiary amine groups, and a flexible hyper-branched structure [11,12]. Therefore, its overall cationic nature and functionality make the PAMAM dendrimer a suitable candidate for gene delivery applications [13,14,15].

**Figure 1:**
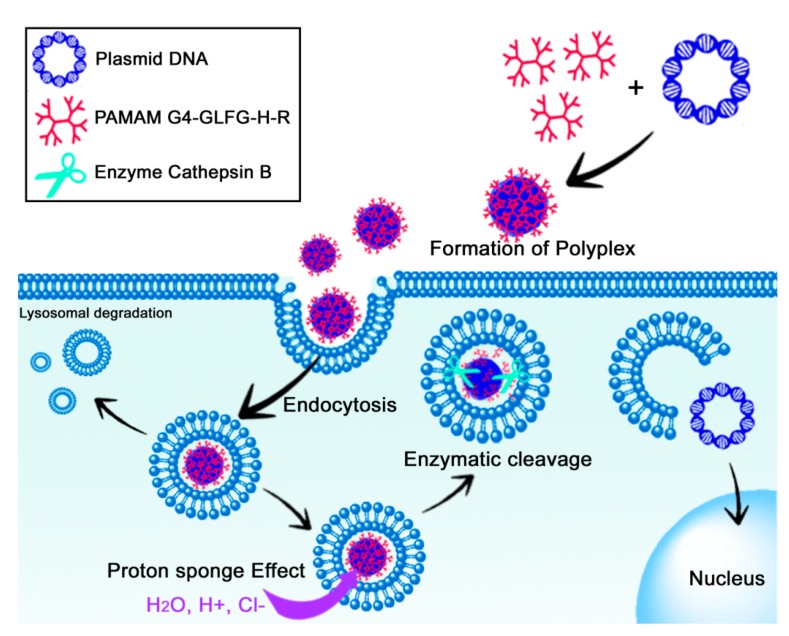
Illustration of endocytosis and gene delivery of poly(amidoamine) (PAMAM) generation 4 (G4)-GLFG-H-R/pDNA polyplex. GFLG: glycine-phenylalanine-leucine-glycine; H: histidine; R: arginine.

However, unmodified PAMAM dendrimers have low transfection efficiency compared with the gold standard polymer PEI 25 kD [16]. Nevertheless, PAMAM dendrimers have recently emerged as potential gene delivery carriers through chemical modification, such as cationic polymers, cancer-targeting moieties, and enzyme-sensitive linkers. Several studies have demonstrated that the modification of cationic polymers results in a high gene transfection efficiency and high cellular uptake based on degradation of the polymers by factors specific to the intracellular environment, such as glutathione (GSH), pH, and peptidase. Previously, our group has reported various attempts to improve the transfection efficiency of PAMAM generation 4 (G4) using single amino acids or short peptide sequences [17,18,19]. In the present work, the cathepsin B-cleavable PAMAM dendrimer G4-GLFG-H-R was synthesized and evaluated for its ability to enhance cellular uptake, endosomal escape, and subsequent transfection efficiency (Figure 1). The GFLG (glycine-phenylalanine-leucine-glycine) sequence is an enzyme-cleavable linker that has previously been reported to promote destabilization in the presence of the lysosomal enzyme cathepsin B [20,21]. G4-GLFG-H-R can be degraded by cathepsin B, releasing pDNA from the polyplex. This enzyme-sensitive property of G4-GLFG-H-R can result in enhanced transfection efficiency compared with unmodified G4. In addition, histidine (H) and arginine (R) were also introduced onto the surfaces of the PAMAM dendrimer containing the GFLG sequence to enhance the proton sponge effect and cellular uptake, respectively [17]. Moreover, cathepsin B was reported to be implicated in tumor progression, and cathepsin B-sensitive G4-GLFG-H-R has potential applications in gene delivery and cancer therapy [22].

## 2. Experimental

### 2.1. Materials

Dulbecco modified eagle medium (DMEM), Dulbecco′s phosphate-buffered saline (DPBS), antibiotic-antimycotic (100×) solution, trypsin-EDTA solution (0.25%), and fetal bovine serum (FBS) were purchased from Gibco (Gaithersburg, MD, USA). Deuterium oxide (D_2_O), 4-(2-hydroxyethyl)piperazine-1-ethanesulfonic acid (HEPES), piperidine, triisopropylsilane (TIS), trifluoroacetic acid (TFA), cathepsin B (from human liver), thiazolyl blue tetrazolium bromide (MTT), ethidium bromide (EtBr), dimethyl sulfoxide (DMSO), dimethylformamide (DMF), PAMAM dendrimer (ethylene diamine core, generation 4.0 solution), and *N*,*N*-diisopropylethylamine (DIPEA) were purchased from Sigma Aldrich (St. Louis, MO, USA). Fmoc-Phe-OH, *N*-α-Fmoc-*N*-*g*-(2,2,4,6,7-pentamethyldihydrobenzofuran-5-sulfonyl(pbf))-l-arginine, Fmoc-Leu-OH, and 1-hydroxybenzotriazole hydrate (HOBt) were obtained from Anaspec (Fremont, CA, USA). Fmoc-His(trt)-OH, Fmoc-Gly-OH, and 2-(1*H*-benzotriazole-1-yl)-1,1,3,3-tetramethyluronium hexaafluorophosphate (HBTU) were purchased from Novabiochem (Darmstadt, Germany). The PicoGreen assay kit was obtained from Invitrogen (Carlsbad, CA, USA). The BCA protein assay kit was purchased from Thermo Fisher Scientific (Hudson, NH, USA). The luciferase assay kit was obtained from Promega (Madison, WI, USA). Spectra/Por7 dialysis tube (MWCO = 3500) was purchased from Spectrum Labs (Rancho Dominguez, CA, USA). Diethyl ether was obtained from JUNSEI (Tokyo, Japan). Agarose was purchased from Lonza (Basel, Switzerland). Reporter lysis buffer was purchased from Promega (Madison, WI, USA).

### 2.2. Synthesis of PAMAM G4-GLFG-H-R

PAMAM G4-GLFG-H-R was synthesized following standard Fmoc chemistry [12]. PAMAM G4 was conjugated by Fmoc-Gly-OH (4 eq.) with HOBt (4 eq.), HBTU (4 eq.), and DIPEA (8 eq.) in DMF/DMSO (1:1, *v*/*v*) solvent for 16 h at room temperature (RT). PAMAM G4-Gly-Fmoc was precipitated by excess cold ethyl ether, collected using a centrifuge and dried using an N_2_ gas stream. For removal of the Fmoc group, G4-Gly-Fmoc was dissolved in 30% piperidine solution (DMF/DMSO:piperidine, 7:3, *v*/*v*) and reacted for 1.5 h at RT in protected light. The products were re-precipitated using excess cold diethyl ether. Following the above synthesis procedure, PAMAM G4-Gly-NH_2_ was conjugated to Fmoc-Leu-OH, Fmoc-Phe-OH, Fmoc-Gly-OH, Fmoc-His(trt)-OH, and Fmoc-Arg (pbf)-OH. To remove the pbf and trt protecting groups on each amino acid unit, the products were dissolved in de-protection reagent (TFA:TIS:water = 90:5:5, *v*/*v*) and reacted for 6 h at RT. The final product PAMAM G4-GLFG-H-R was dialyzed using a dialysis membrane (MWCO, 3500) in distillated water. After the dialysis procedure, the product was obtained using a freeze dryer. The control group, PAMAM G4-GLFG-R was conjugated without histidine using the above mentioned procedure. The structure of the product was confirmed using 300 MHz ^1^H-NMR spectroscopy (Fourier 300, Bruker, Billerica, MA, USA) in deuterium water (D_2_O).

### 2.3. Gel Retardation Assay and PicoGreen Assay

A gel retardation assay was performed using an agarose gel. Polyplexes of pDNA and polymer were prepared at various weight ratios (polymer (0.25, 0.5, 1, 2, 2.5 μg):pDNA, *w*/*w*) and incubated in HEPES buffer (25 mM, pH 7.4) for 30 min. Prepared samples were loaded onto 0.7% agarose gel with ethidium bromide. Agarose gel electrophoresis was performed for 30 min at 100 V. The PicoGreen assay was performed to confirm the polyplex formation ratio by measuring fluorescence intensity. Measurement of PicoGreen was prepared according to the manufacturer’s protocol. Polyplexes were incubated with pDNA and G4-GLFG-R or G4-GLFG-H-R (1, 4, 8, 10, and 15 µg, polymer:pDNA, *w*/*w*) in 25 mM HEPES buffer for 30 min. Sample was mixed in TE buffer of 800 µL (10 mM Tris-HCl, 1 mM ethylenediaminetetraacetic acid (EDTA), pH 7.4), and sample was incubated with TE buffer containing PicoGreen reagent of 2 µL for 2 min. Then, sample was measured at an excitation wavelength of 480 nm and emission wavelength of 520 nm using a fluorometer (Quantech Base Model Filter Fluorometer, Thermo Fisher Scientific, Hudson, NH, USA).

$$\mathrm{Calculation}\;\mathrm{of}\;\mathrm{fluorescence}\;\mathrm{ratio}\;(\%)=\frac{F_{e}-F_{b}}{F_{p}-F_{b}}\times 100$$

In the formula, *F*_e_, *F*_p_, and *F*_b_ represent the fluorescence intensity of experimental sample, control (plasmid DNA), and blank, respectively.

### 2.4. Measurement of Dynamic Light Scattering (DLS) and Zeta Potential

The size distribution and zeta potential of polyplexes were measured using DLS and potential analysis. Polyplexes of G4-GLFG-R and G4-GLFG-H-R were prepared at 8:1 (polymer (5 μg):pDNA) and 10:1 *w*/*w* ratio. PEI 25 kD and PAMAM G4 were prepared at 3:1 (polymer (1.5 μg):pDNA) and 4:1 (polymer (2 μg):pDNA) *w*/*w* ratio. Naked pDNA (pCN-luc gene, 0.5 μg) was used a control group. Polymer and pDNA were incubated for 30 min in 25 mM HEPES buffer and diluted with distilled water (final volume 1 mL). The size and zeta potential were then measured using DLS (ELS-Z, Otsuka Electronics, Osaka, Japan) and zeta instrument (Malvern, London, UK), respectively. All samples were analyzed at room temperature, and measurements were repeated three times.

### 2.5. Acid–Base Titration Assay

To evaluate buffering capacity, pH values of G4-GLFG-R and G4-GLFG-H-R were determined by the acid–base titration method [17]. PEI 25 kD (2 mg, 8 × 10^−8^ M), PAMAM G4, G4-GLFG-R, and G4-GLFG-H-R were prepared using the same equivalents. Each sample was prepared in 4 mL of 150 mM NaCl solution and 100 µL of 1 N NaOH solution and NaCl was used as the control group. Samples were titrated using 20 µL of 0.1 N HCl solution until pH 3.0 was reached. The pH values of the samples were measured with a pH-meter (pH 211 microprocessor pH meter, HANA Instruments, Seoul, Korea).

### 2.6. Enzymatic Release Test

The in vitro enzymatic release behaviors of plasmid DNA from G4-GLFG-H-R polyplexes triggered by the enzyme cathepsin B were investigated using agarose gel electrophoresis. Polyplexes of G4-GLFG-H-R and PAMAM G4 were prepared at 8:1 (polymer (2 µg):pDNA) and 4:1 (polymer (1 µg):pDNA) weight ratio in D.W. Cathepsin B was prepared as previously reported [20]. Briefly, cathepsin B was dissolved in 0.1 M acetate buffer (pH 5.0) containing 0.01 M EDTA and 0.05 M reduced GSH (working buffer) to a final concentration of 0.5 µM. Samples were incubated with cathepsin B for 1, 2 and 4 h at 37 °C in a water bath, and control polyplex was incubated in working buffer without cathepsin B. The incubated samples were analyzed by 0.7% agarose gel.

### 2.7. Cell Culture and Cytotoxicity Assay of Polymer and Polyplex

HeLa human epithelial carcinoma cells (ATCC^®^ CCL-2), and L929 mouse fibroblasts (ATCC^®^ CCL-1) were maintained in a CO_2_ incubator (37 °C, 5% CO_2_). HeLa cells were grown in 89% Dulbecco’s modified Eagle’s medium (DMEM) with 10% fetal bovine serum (FBS) and 1% antibiotic-antimycotic solution and L929 cells were grown in 89% RPMI 1640 with 10% FBS and 1% antibiotic-antimycotic solution. Cells were subcultured using 0.25% trypsin-EDTA solution. Cytotoxicity assays were performed using MTT (3-(4,5-dimethylthiazol-2-yl)-2,5-diphenyl -tetrazolium bromide) in HeLa, L929 cells. Cells were seeded at a density of 1.5 × 10^4^ cells/well in 96-well plates. Cells were then treated with G4-GLFG-R or G4-GLFG-H-R at final concentrations 12.5, 25, 50, 100 and 200 µg/mL. After 24 h, 10 µL MTT solutions (2 mg/mL in DPBS) were added to each well, and cells were incubated for an additional 4 h. After incubation, old medium was removed and formazan was dissolved in DMSO. Absorbance was measured at 570 nm using a VERSAmax microplate reader (Molecular Devices, Sunnyvale, CA, USA). A cytotoxicity assay of polyplex was performed using MTT assay in L929 cells. Cells were seeded at a density of 1.5 × 10^4^ cells/well in 96-well plates. Polyplex was prepared PEI 25kD (3:1, polymer:pDNA (0.2 µg), *w*/*w*), PAMAM G4 (4:1), G4-GLFG-R (10:1), and G4-GLFG-H-R (10:1), and sample was treated. After 24 h, MTT solutions were added to each well and the above procedure was carried out.

$$\mathrm{Calculation}\;\mathrm{of}\;\mathrm{cell}\;\mathrm{viability}\;(\%)=\frac{A_{e}-A_{b}}{A_{c}-A_{b}}\times 100$$

In the formula, *A*_e_, *A*_b_, and *A*_c_ represent the absorbance of experimental sample, control (cell only), and blank, respectively.

### 2.8. Confocal Microscopy

HeLa cells (1.0 × 10^4^ cells/well) were seeded on µ-Slide 8 well (Ibidi) and incubated at 37 °C. After 24 h incubation, the cells were treated with polyplexes composed of various polymers. Polymers were labeled with Alexa Fluor 488 (Green), and pDNA was labeled with Alexa Fluor 546 (Red). The labeling samples were prepared according to the manufacturer’s protocol. After further incubation for 24 h, the old medium was removed, and the cells were washed with PBS. Nuclei were dyed with Hoechst 33342. The fluorescent signal was analyzed using a Zeiss LSM 5 Live confocal laser microscope (Zeiss, Oberkochen, Germany).

### 2.9. Transfection Assay

The transfection efficiency of the polymer was investigated by luciferase assays. Plasmid reporter gene (pCN-luc) was prepared as reported previously [23]. HeLa cells were seeded at a density of 4 × 10^4^ cells/well in a 24-well plate. Polymer and pDNA were formed polyplex and then mixed with cell media solution. Samples were treated with the polyplex (PEI 25 kD (3:1), PAMAM G4 (4:1) G4-GLFG-R and G4-GLFG-H-R (10:1 and 20:1), polymer:pDNA (1 µg)) and incubated for 24 h. Cells were removed from the old medium and washed twice using DPBS. Cells were treated with reporter lysis buffer and incubated for 20 min. The cell lysates were harvested and centrifuged at 13,200× *g* for 10 min, and supernatants were collected. The protein concentration was quantified using a MicroBCA Protein Assay Kit. The luciferase activity of the sample was measured using a luminometer (Lumat LB 9507, Berthold Technology, Bad Wildbad, Germany). Transfection efficiency was calculated as relative luciferase units (RLU)/amount of protein.

### 2.10. Statistical Analysis

Differences between groups were considered to be statistically significant at *p* < 0.01 (**) and *p* < 0.00l (***). The statistical analysis was performed using an unpaired Student′s *t*-test (Graph Pad Prism5).

## 3. Result and Discussion

### 3.1. Synthesis and Characterization

**Figure 2:**
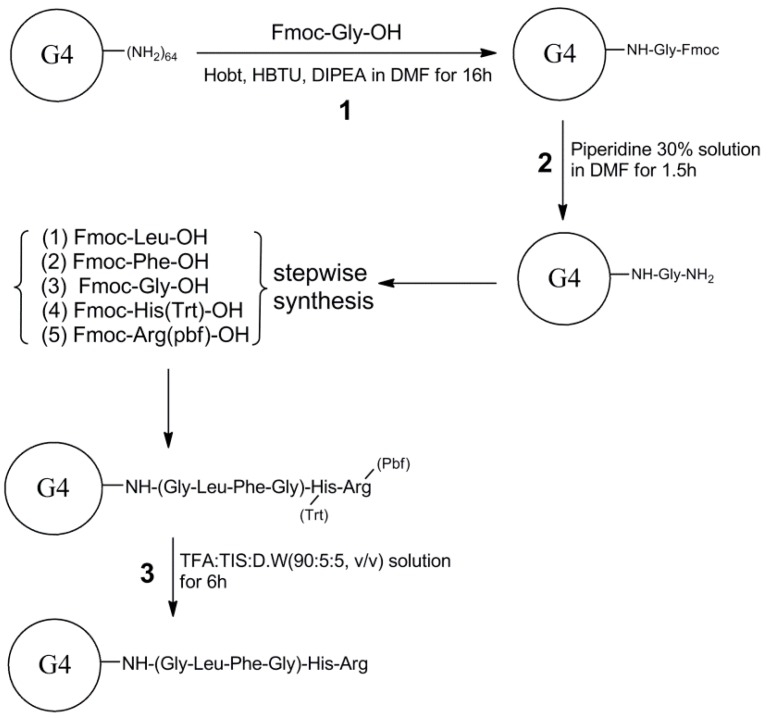
Synthesis scheme of PAMAM G4-GLFG-H-R. DIPEA: *N*,*N*-diisopropylethylamine; DMF: dimethylformamide; HBTU: 2-(1*H*-benzotriazole-1-yl)-1,1,3,3-tetramethyluronium hexaafluoropho-sphate; HOBt: 1-hydroxybenzotriazole hydrate; TFA: trifluoroacetic acid; TIS: triisopropylsilane.

**Figure 3:**
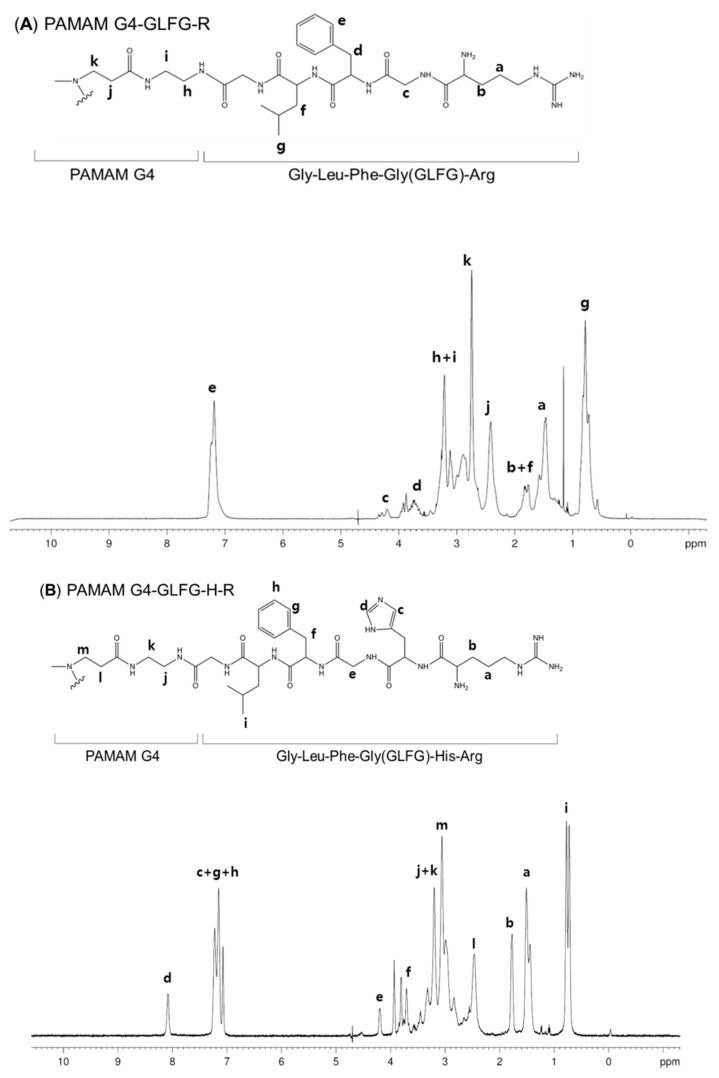
^1^H NMR data of (**A**) PAMAM G4-GLFG-R and (**B**) G4-GLFG-H-R.

We synthesized the enzyme-sensitive carrier PAMAM G4-GLFG-H-R using the Fmoc chemistry method. The general procedure for G4-GLFG-H-R synthesis is shown in Figure 2. The surface amine of PAMAM G4 was reacted with the carboxylic group of the peptide. The final products were measured using 300 MHz ^1^H-nuclear magnetic resonance, and the results are shown in Figure 3.

PAMAM G4-GLFG-R: δ (ppm) 0.78 (–CH(C*H*_3_)_2_CHCH_2_CH–) of leucine, 1.47 (–HCCH_2_C*H*_2_CH_2_NH–), 1.82 (–HCC*H*_2_CH_2_CH_2_NH–) of arginine, 2.41 (–NHCH_2_C*H*_2_CO–), 2.744 (–NHC*H*_2_CH_2_CO–), 3.12 (–NHC*H*_2_CH_2_NH–), 3.22 (–NHCH_2_C*H*_2_NH–) of the PAMAM G4 units, 3.65 (–COCH*CH*_2_(C_6_H_5_)NH–) of phenylalanine, 4.20 (–*H*CCH_2_CH_2_CH_2_NH) of arginine, 7.19 (–COCHCH_2_(C_6_*H*_5_)NH–) of phenylalanine (Figure 3A).

PAMAM G4-GLFG-H-R: δ (ppm) 0.77 (–CH(C*H*_3_)_2_CHCH_2_CH–) of leucine, 1.51 (–HCCH_2_C*H*_2_CH_2_NH–) and 1.89 (–HCC*H*_2_CH_2_CH_2_NH–) of arginine, 2.56 (–NHCH_2_C*H*_2_CO–), 3.06 (–NHC*H*_2_CH_2_CO–) and 3.20 (–NHCH_2_C*H*_2_NH–) of the PAMAM G4 units, 4.19 (–*H*CCH_2_CH_2_CH_2_NH) of arginine, 7.07, 7.22 (–COCHCH_2_(C_6_*H*_5_)NH–) of phenylalanine, 7.15 (–CH(CH_2_(CC*H*NCHNH))NH–), and 8.08 (–CH(CH_2_(CCHNC*H*NH))NH–) of histidine (Figure 3B).

### 3.2. Gel Retardation Assay and PicoGreen Assay

**Figure 4:**
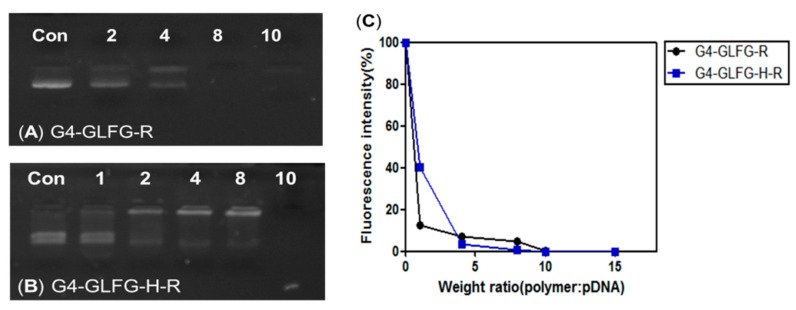
(**A**,**B**) Agarose gel retardation and (**C**) PicoGreen reagent assay of polyplexes.

Generally, the positive charge of cationic polymer is an important factor for the condensation of pDNA and the formation of stable polyplexes. Since both G4-GLFG-R and G4-GLFG-H-R possess peripheral arginine units, they could contribute to effective complex formation with pDNA by electrostatic interaction. The pDNA binding ability of G4-GLFG-R and G4-GLFG-H-R were examined by gel retardation assay. The G4-GLFG-R/pDNA and G4-GLFG-H-R/pDNA polyplexes were prepared at various polymer weight ratios, and the pDNA band was confirmed by agarose gel electrophoresis. Notably, pDNA migration decreased as the amount of G4-GLFG-H-R increased, and the pDNA band completely disappeared at a polymer weight ratio of 10:1 (Figure 4B). This result demonstrated that G4-GLFG-R was complexed with pDNA at weight ratio of 8:1 (Figure 4A), and the polyplex formation of G4-GLFG-H-R was observed at higher polymer/pDNA weight ratio (10:1, *w*/*w*) compared with G4-GLFG-R (8:1, *w*/*w*). These findings may be explained by the differences in molecular weight and surface charge density of G4-GLFG-R and G4-GLFG-H-R. Since G4-GLFG-H-R has histidine residues having imidazole groups as side chains, these groups could be poorly protonated at pH 7.4. Therefore, G4-GLFG-H-R has a lower surface charge density compared to G4-GLFG-R, and a greater amount of polymer may be required for complete polyplex formation.

Additionally, the polyplex formation of G4-GLFG-H-R was precisely evaluated by PicoGreen reagent assay [24], which can determine polyplex formation via the sensitive interactions with PicoGreen probe in the aqueous phase. Polyplex of G4-GLFG-H-R with pDNA was detected at an excitation wavelength of 480 nm and emission wavelength of 520 nm using a fluorometer (Figure 4C). The fluorescence intensity ratio decreases due to increasing polymer amount, indicating that both G4-GLFG-R and G4-GLFG-H-R could form mature polyplexes with pDNA at a 10:1 weight ratio. Accordingly, we investigated polyplexes prepared at 10:1 weight ratio for subsequent assays.

### 3.3. Diameter and Zeta Potential of Polyplexes

**Figure 5:**
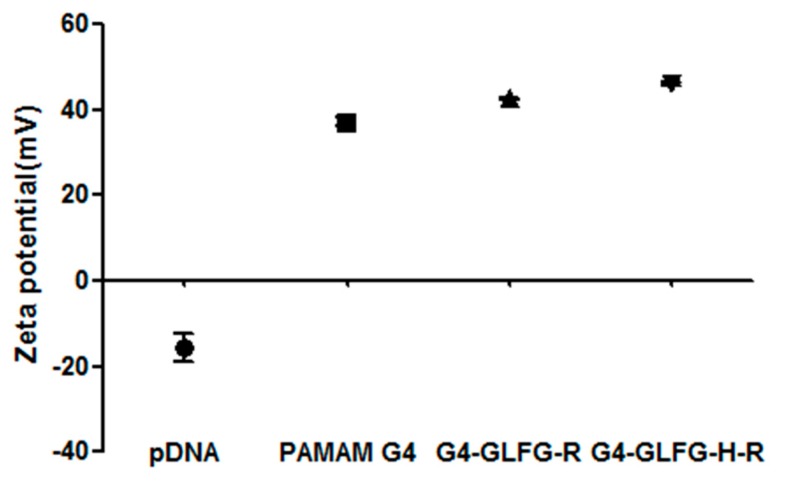
Zeta potential graph of polyplexes.

For stable gene transfection into cells, polyplex should overcome unwanted reactions such as nuclease action and endosomal/lysosomal degradation during cellular pathway [25]. Therefore, the formation of stable and compact polyplexes is essential for releasing and protecting pDNA. The formation of a cationic polyplex allows the anions of the cell membrane to be overcome and facilitates the stable release of pDNA into the cytosol. The diameter and surface charge of G4-GLFG-R and G4-GLFG-H-R was analyzed by DLS and zeta potential measurements. As shown in Table 1, the polyplex of G4-GLFG-R:pDNA (8:1, *w*/*w*) showed a mean diameter of 199.3 ± 3.9 nm, and G4-GLFG-H-R:pDNA (10:1, *w*/*w*) was 177.23 ± 2.6 nm. Both polyplexes displayed low polydispersity index (PDI), indicating that these polyplexes were homogenous. This result indicated that these PAMAM derivatives can form nano-scale polyplexes owing to electrostatic interactions. The small size of the polyplexes could facilitate effective cellular uptake for gene delivery. As shown in Figure 5, native PAMAM G4/pDNA showed a positive charge of 37.2 ± 1.95 mV and polyplexes of G4-GLFG-R and G4-GLFG-H-R also showed positively charged zeta potential values of 42.63 ± 0.38 mV and 46.03 ± 0.81 mV, respectively. The polyplexes of G4-GLFG-R and G4-GLFG-H-R have values of more than 20 mV, and are thought to possess sufficient cationic charge to be delivered inside cells.

### 3.4. Acid–Base Titration

**Figure 6:**
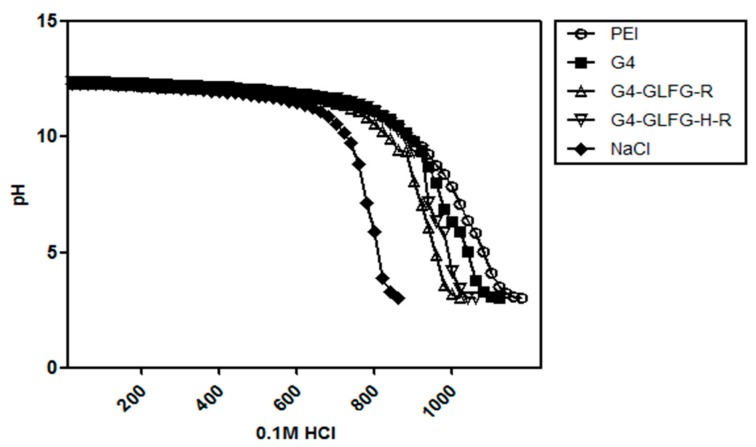
Acid–base titration of G4-GLFG-R and G4-GLFG-H-R.

G4-GLFG-H-R has an imidazole group from the histidine residue, which has a p*K*a value of about 6 and is mostly protonated at acidic pH conditions less than 6. Thus, G4-GLFG-H-R is believed to have a proton buffering effect at endolysosomal pH values, and G4-GLFG-H-R can play an important role in intracellular escape based on the buffering capacity effect [17]. As shown in Figure 6, as a positive control, PEI 25 kD showed strong buffering capacity effect. Additionally, PAMAM G4, G4-GLFG-R, and G4-GLFG-H-R showed proton buffering effect compared to NaCl control. As expected, G4-GLFG-H-R showed increased buffering capacity at pH 5–7 compared to G4-GLFG-R, suggesting that G4-GLFG-H-R could overcome endosomal acidic conditions and might be more effective at gene transfection efficiency.

### 3.5. Plasmid DNA Release Test by Enzyme Cathepsin B

G4-GLFG-H-R has the enzyme-sensitive sequence Gly-Phe-Leu-Gly (GFLG), specific for cleavage by cathepsin B. Cathepsin B is localized to the cytoplasm and endo/lysosome compartment [26,27]. The environment of the lysosome is at pH 4.5–5.0, and application of a GFLG linker can induce the effective release of cargo molecules. Hence, we analyzed pDNA release from the polyplex by treatment with cathepsin B at pH 5.

**Figure 7:**
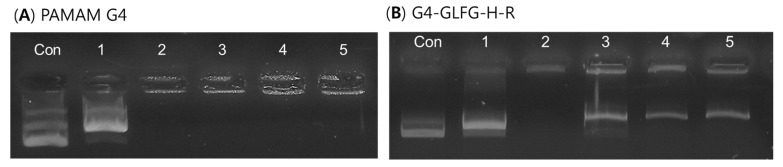
Plasmid DNA release test by cathepsin B, pDNA(control), pDNA + Cathepsin B (lane 1), Polymer:pDNA polyplex (G4-GLFG-H-R, 10:1, PAMAM G4, 4:1, *w*/*w*) (lane 2), polyplex + cathepsin B incubated for 4 h (lane 3), polyplex + cathepsin B incubated for 2 h (lane 4), polyplex + cathepsin B incubated for 1 h (lane 5).

The G4-GLFG-H-R/pDNA polyplex was treated with cathepsin B and release of pDNA was detected using electrophoresis in vitro. As shown in Figure 7B, naked pDNA was used as a control. Lane 1 shows pDNA with cathepsin B, which has negligible effects on pDNA. Lane 2 shows polyplexes of G4-GLFG-H-R without cathepsin B, it can confirm the formation of a stable polyplex. Lanes 3–5 show pDNA released from the polyplex of G4-GLFG-H-R by cathepsin B. Lanes 4 and 5 show similar amounts of released pDNA by cathepsin B treatment for 1–2 h, and a prominent band of pDNA confirmed at 4 h compared with 1 h and 2 h time points.

However, as shown in Figure 7A, native PAMAM G4/pDNA polyplex could not release any pDNA by cathepsin B treatment under the same conditions. These results demonstrated that the cathepsin B-sensitive PAMAM dendrimer G4-GLFG-H-R could release the cargo molecule via specific enzyme [28]. This enzyme-sensitive ability could contribute to enhanced transfection efficiency in cells.

### 3.6. Cytotoxicity Assay

**Figure 8:**
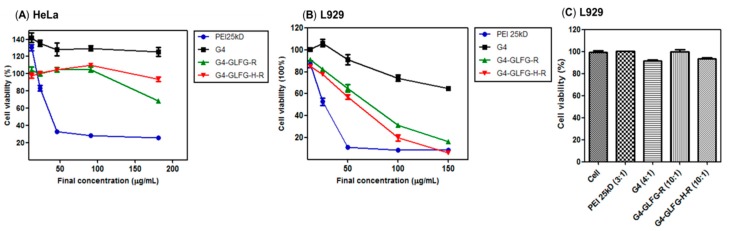
MTT (3-(4,5-dimethylthiazol-2-yl)-2,5-diphenyltetrazolium bromide) assay in HeLa and L929 cells. (**A**,**B**) Cytotoxicity test depending on polymer concentration; (**C**) cytotoxicity test of polyplex in L929, polymer:pDNA, *w*/*w*. Data are shown as mean ± standard deviation (*n* = 3).

Many efficient gene delivery carriers have a strong positive charge, thereby improving cellular uptake and transfection efficiency. However, excess positive charge can cause cell cytotoxicity due to the strong interaction of cell membrane components, as is seen with PEI 25kD [29,30]. The cytotoxicity of G4-GLFG-H-R was confirmed with various polymer concentrations using MTT assays in HeLa and L929 cells. To evaluate the cytotoxicity of the polymer, PEI 25kD, G4, and G4-GLFG-R were compared with G4-GLFG-H-R in HeLa cells. As shown in Figure 8A, PEI 25 kD showed high cytotoxicity, whereas PAMAM G4, G4-GLFG-R, and G4-GLFG-H-R were relatively nontoxic at various concentrations. To further evaluate the polymer-mediated toxic effect at primary cells, cytotoxicity was assessed using L929 mouse primary cells (Figure 8B). PAMAM G4 showed a slight toxicity at high concentrations, and the G4-GLFG-R as well as G4-GLFG-H-R also displayed significant and dose-dependent cytotoxicity at high concentration levels (over 50 µg/mL) in L929 cells. Although severe toxicity was observed at high polymer concentrations, the usual concentration levels for polyplex-mediated transfection are much less than the concentration ranges which showed cytotoxicity. To address this issue, we further evaluated cytotoxicity test of polyplexes in L929 cells (Figure 8C). Cytotoxicity was evaluated with PEI 25 kD/pDNA (3:1, polymer:pDNA, *w*/*w*), PAMAM G4/pDNA (4:1), G4-GLFG-R/pDNA (10:1), and G4-GLFG-H-R/pDNA (10:1), respectively. As expected, no polyplexes showed any cytotoxic effect for the cells at the given concentration levels.

### 3.7. Confocal Microscopy

**Figure 9:**
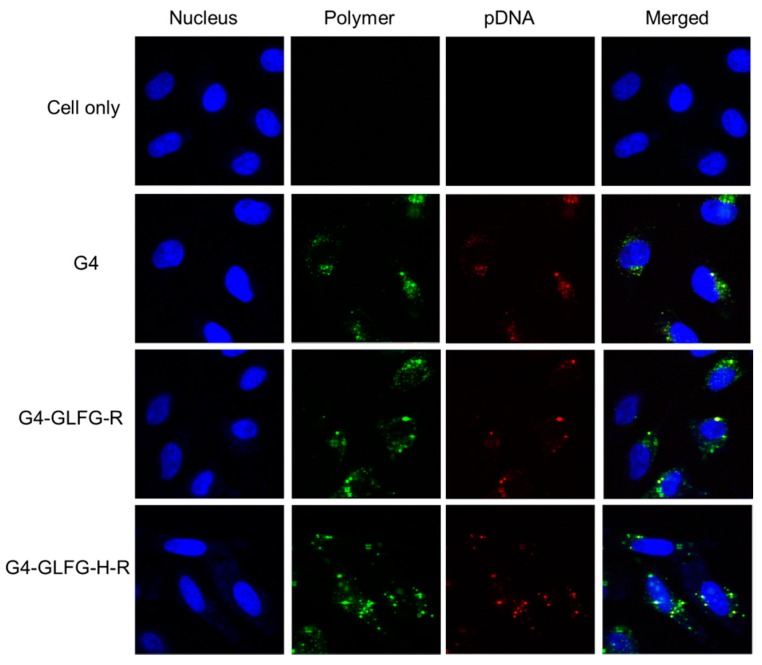
Cellular uptake using confocal microscopy in HeLa cells.

To observe cellular internalization of G4-GLFG-H-R, the polymer was labeled with Alexa Fluor 488 (Green), and pDNA was labeled with Alexa Fluor 546 (Red). The labeled polyplexes were incubated with HeLa cells for 24 h and observed using confocal microscopy. As shown in Figure 9, PAMAM G4/pDNA polyplex exhibited red and green fluorescence signal in the cytoplasm, andG4-GLFG-R/pDNA and G4-GLFG-H-R/pDNA polyplexes showed overall distribution inside the cells. Interestingly, the pDNA signal of G4-GLFG-H-R polyplexes were mostly localized in the perinuclear sites compared to other polyplexes. These results indicate that G4-GLFG-H-R facilitates endosomal escape, and pDNA could be effectively released into cytoplasm owing to the GFLG sequence by cathepsin B.

### 3.8. Transfection Assay

**Figure 10:**
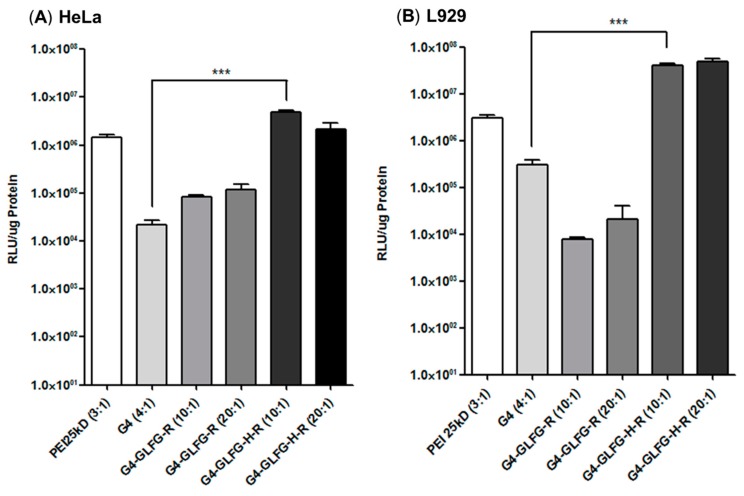
Transfection assay using pCN-luc reporter gene in HeLa (**A**) and L929 (**B**) cells. Data are shown as mean ± standard deviation (*n* = 3). *** *p* < 0.001.

To evaluate transfection efficiency, firefly luciferase gene (pCN-luc gene) was used as a reporter gene and luciferase activity was detected from isolated protein in HeLa and L929 cells. Polyplexes of G4-GLFG-R and G4-GLFG-H-R were prepared at both 10:1 and 20:1 weight ratios. Branched PEI 25 kD and PAMAM G4 dendrimers were used as the control. Interestingly, as shown in Figure 10, G4-GLFG-H-R displayed higher transfection efficiency compared to PAMAM G4 and PEI 25 kD for HeLa and L929 cells. The buffering capacity of G4-GLFG-H-R may induce the release of the polyplexes from endo/lysosomes and increase the gene expression level due to plasmid DNA release by cathepsin B [17]. Finally, G4-GLFG-H-R has enhanced transfection efficiency owing to efficient intracellular uptake, increased proton buffering capacity, and enzyme-sensitive collapse of polyplexes.

## 4. Conclusions

In this study, PAMAM G4 was conjugated with histidine, arginine, and the enzyme-sensitive sequence “GFLG”. Polyplexes of G4-GLFG-H-R/pDNA were formed nano-sized and positively charged using DLS and zeta potential measurements. Additionally, the cytotoxicity of G4-GLFG-H-R was evaluated and transfection assays were performed in HeLa and L929 cells. G4-GLFG-H-R promoted high cell viability in HeLa cells and showed high transfection efficiency in HeLa and L929 cells due to the enzyme-cleavable GFLG linker and the buffering capacity effect of histidine. Finally, confocal microscopy results confirmed the effective delivery of pDNA in the cytoplasm and nucleus. So, PAMAM G4-GLFG-H-R could be a potential gene and drug delivery carrier for biotechnological applications.

## Figures and Tables

**Table 1:** Zeta potential and mean diameter values of polyplexes.

Sample	Zeta Potential	Diameter ^a^	Polydispersity (PDI) ^a^
pDNA	–15.46 ± 5.69	-	-
PAMAM G4	+37.20 ± 1.95	237.0 ± 6.3	0.07
G4-GLFG-R	+42.63 ± 0.38	199.3 ± 3.9	0.17
G4-GLFG-H-R	+46.03 ± 0.81	177.2 ± 2.6	0.17

^a^ Determined using dynamic light scattering (DLS) measurements at room temperature (RT); measurements were repeated three times.
